# The Performance of Gram-Staining in Tailoring the Empirical Antibiotic Choice in Operated Diabetic Foot Infections

**DOI:** 10.3390/jcm14072468

**Published:** 2025-04-04

**Authors:** Dominique Altmann, Jonas Liebe, Felix W. A. Waibel, Madlaina Schöni, Francesca Napoli, Christina Sydler, Fabian Schläfli, Linus Ledermann, Benjamin A. Lipsky, Ilker Uçkay

**Affiliations:** 1 Technical Orthopedics and Neuro-Orthopedics Team, Department of Orthopedic Surgery, Balgrist University Hospital, University of Zurich, 8008 Zurich, Switzerlandfelix.waibel@balgrist.ch (F.W.A.W.);; 2 Department of Medicine, University of Washington, Seattle, WA 98195, USA; 3 Infectious Diseases, Balgrist University Hospital, University of Zurich, 8008 Zurich, Switzerland

**Keywords:** diabetic foot infections, gram stain, microbiological tissue cultures, prediction, pathogen groups, antibiotic stewardship, pilot evaluation

## Abstract

**Background/Objectives:** Current international guidelines recommend choosing an empirical antibiotic regimen for treating diabetic foot infections (DFI) based largely on clinical severity of the infection and local microbiological epidemiology. This may lead to selecting unnecessarily broad-spectrum initial empiric antibiotic therapy. **Methods:** Using data from our hospital in a large Swiss city, we retrospectively analyzed the performance of the Gram-stained smears of predominantly deep surgical DFI specimens processed by our microbiology laboratory in predicting the microorganism grown on standard cultures. We excluded episodes with paucibacillary stain results, which we interpret as contamination. **Results:** Among 1235 operated moderates or severe DFIs, Gram-stained smear was reported in 321 (26%) of cases, and showed bacteria in 172 episodes (54%) of these. Overall, among Gram stain results with organism seen, the sensitivity, specificity, accuracy, positive and negative predictive values of the Gram stain smear when compared with the cultures was 56%, 93%, 97%, and 38%, respectively. The accuracy was 73%. The corresponding statistical values specifically for Gram-negative bacteria were 61%, 97%, 50%, and 82%. **Conclusions**: The results of routine Gram stain smears of deep intraoperative DFI specimens generally lack sufficient sensitivity, and was only useful to reasonably exclude a DFI caused predominantly by Gram-negative bacteria. For Gram-stained smears results to be useful for guiding antibiotic stewardship, we need prospective trials to assess their value in different types of DFIs.

## 1. Introduction

The most recent guidelines of the International Working Group on the Diabetic Foot (IWGDF) [1] and the Infectious Diseases Society of America (IDSA) [2] recommend that the initial (empirical) antibiotic regimen for diabetic foot infections (DFI) should be based on the results of optimally obtained wound cultures (deep tissue, if available) and the clinical severity of infection. In geographical regions with limited laboratory or surgical facilities [3,4] and a high prevalence of antibiotic-resistant bacteria (often gram-negative rods, such as *Pseudomonas* spp.) [5], it is wise to prescribe an initial broad-spectrum regimen to avoid failing to adequately cover pathogens in a rapidly evolving infection. Having a point of care test that could rapidly help predict the likely culture results could help guide (and often constrain) the selection of antibiotic agents.

One such test could be the Gram-stained smear of wound specimens. This could, at a minimum, help to tailor the antibiotics to those active against Gram-positive pathogens only, or to add those active against Gram-negative pathogens as well. The Gram-stained smear is an easily performed and inexpensive test that has been used for this purpose for over 100 years and in various infectious scenarios [6]. It can detect unusual pathogens, help distinguish between contaminated and well-obtained specimens and might partially replace other more expensive microbiological diagnostic procedures that take several days to provide results [6]. These advantages can make using the Gram-stained smear an important part of antibiotic stewardship programs [4,5], especially by reducing unnecessary, costly and often intravenous antibiotic coverage in settings already marked by a high prevalence of antibiotic-resistant microorganisms and limited resources.

Published data on the value of the Gram-stained smear for soft tissue wounds, including DFI, are limited. In a study in Tanzania, Abbas et al. probed deep at the base of infected diabetic foot ulcers and aseptically collected two deep tissue specimens with a sterile scalpel [3,4]. They then performed Gram-stained smears and standard bacterial cultures on the specimens. Among 128 specimens, 118 (92%) yielded bacterial growth, among which there was 96.4% congruency between the Gram stain and microbiological culture results [3]. Of note is that the Gram stains correctly predicted the presence of Gram-negative organisms in 82% of cases. Similarly, a study in Japan by Taniguchi et al., demonstrated that among 208 patients Gram stain smears of respiratory, urinary, or skin and soft tissues specimens performed by healthcare professionals directly contributed fewer broad-spectrum antibiotics being prescribed, with 90% of the Gram stain-based treatments judged as effective [7,8,9].

Based on the results of a narrative review, the authors of a recent opinion paper [4] called for efforts to evaluate the potential value of the Gram stain smear as part of antibiotic stewardship programs for managing DFIs, with special emphasis on less developed, limited resources areas. Resource-rich areas are facing an ever-increasing prevalence of antibiotic resistance among Gram-negative pathogen groups [4,5,10,11]. The conditions are, however, still less dire than in less-developed countries, such as Tanzania [3], where access to microbiological cultures is less available, and the prevalence of naturally-resistant Gram-negative rods is higher [12]. In Central and Northern Europe, as in Northern America, the prevalence of Gram-negative rods, including *P. aeruginosa*, as pathogens in community-acquired DFIs oscillates between about 7% and 12% [13]. In contrast to the developing world, at presentation for surgical assessment for a DFI many patients are already receiving empirical antimicrobial therapy prescribed by their general practitioner [14,15,16], which reduces the likelihood of detecting pathogens in Gram-stained smears.

Of note is that the time window for the Gram stain smear providing information about the presence of gram-negative pathogens that can enhance antimicrobial stewarding is quite limited. In patients with a moderately severe DFI undergoing surgical debridement, clinicians generally do not need to provide antibiotic coverage for all potential bacteria. In the absence of sepsis, the therapeutic effect of surgery is so profound that even an apparently incorrect initial empirical antimicrobial regimen does not alter the outcomes [16]. In these resource-rich settings, however, results of a Gram stain smear could reduce the use of unnecessary broad-spectrum empirical therapy in non-severe DFIs [9], while supporting its use in severe DFIs with sepsis [17]. The key issue regarding antibiotic selection is principally about covering antibiotic-resistant gram-negative rods, as virulent gram-positive pathogens (e.g., beta-hemolytic streptococci or *Staphylococcus aureus*) are generally covered by the standard empirical narrow-spectrum agents [1,2], with the exception of methicillin-resistant *S. aureus* (MRSA), the prevalence of which is currently declining globally [4,5,10,11].

In this pilot study, we aimed to retrospectively assess the theoretical potential of the employing routine Gram-stained smears in tailoring a “semi-empirical” antibiotic prescribing in an effort to improve antibiotic stewardship [18]. We sought to determine how accurate the Gram stain smear was in predicting the main pathogen groups in monomicrobial and operated polymicrobial DFIs, including for *P. aeruginosa*, requiring immediate antibiotic treatment. We concentrated our evaluation on moderate and severe DFIs, as these are most likely to require urgent and appropriate antibiotic selections.

## 2. Materials and Methods

### 2.1. Setting, Database, Inclusion and Exclusion Criteria

The Balgrist University Hospital is a referral orthopedic center for diabetic foot problems. From 1 January 2000 to 11 December 2018, we included all adult DFI patients with (partial) lower extremity amputations or surgical debridement in our cohort. Exclusion criteria were: follow-up time less than six months; treatment with major amputation; or, orthopedic implant-related DFIs. For this analysis, the first author (D.A) re-assessed our DFI cohort and focused on Gram-stained smears. Microbiologically, we used Gram stain and culture results conducted on intraoperative (or exceptionally deep tissue) samples [16]. In our hospital we attempt to treat these infections with oral (as opposed to parenteral) antibiotic agents as soon as possible and to target only the “prevalent” or “virulent” pathogen groups in polymicrobial culture results of soft tissue infections.

### 2.2. Statistical Analyses

We analyzed only DFI episodes for which there were visible bacteria on a wound Gram-stained smear. We elected not to include a non-contributive Gram stain attempt within the entire DFI cohort, because the lack of bacterial visualization clinically depends on numerous clinical and laboratory factors that could considerably bias our performance testing on germ identification [6]. For each culture, we used descriptive statistics to compare the Gram stain results with microbiological culture reports and compared groups using the Pearson-χ^2^-test. Specifically, we computed sensitivity, specificity, accuracy, positive and negative predictive values of the Gram stain regarding microbiological cultures [19]. Of note, while sensitivity and specificity are inherent to the performance of the Gram stain, independently of the epidemiological circumstances, the predictive values and the accuracy strongly depend on the local individual prevalence in the patient population [19]. We used STATA™ software (Version 15; College Station, TX, USA).

## 3. Results

### 3.1. Study Population and General Antibiotic Treatment

Among patients in our cohort we analyzed 1,235 moderate or severe DFI cases among adult patients. Overall, 86% of DFIs involved the forefoot, 8% the midfoot, and 3% the hindfoot (among these, 2% were in the calcaneus). The median number of previous diabetic foot surgeries was 1 (range, 1–5 interventions). Overall, 141 DFI episodes (11%) were complicated by Charcot neuroarthropathy. The case-mix of the co-morbidities was large and included 266 females (22%); 927 (75%) under insulin medication; 700 (57%) active smokers with a median of 35 pack-years (interquartile range, 20–60 p-y); 302 (24%) with heavy alcohol consumption; 265 (21%) with ischemic and hypertensive heart diseases and 624 (51%) with chronic renal insufficiency, of which 105 patients (9%) were in renal dialysis; 29 (2%) with organ transplantations, 76 (6%) under immune-suppressive therapy, among which 20 cases were under oncologic chemotherapy [15]. Diabetes was type II in 84% and 54% of all operated cases underwent lower extremity revascularization, of which 44 (3.6%) by surgical lower extremity bypass.

Empirically selected preoperative antibiotic therapy was prescribed by the patients’ general practitioner in 912 (73.9%) of the operated episodes, for a median duration of 13 days preoperatively, using 59 different regimens and dosing schemas [15]. The most frequently used preoperative agents were co-amoxiclav (390/912; 43%) and empiric levofloxacin (26%) [15]. Our initial choice of post-surgical antibiotic regimen did not cover all causative pathogens in 32% of DFI episodes [16]. Routine operative wound care was performed by nurses on the ward for inpatients and by professional wound nurses in the outpatient setting. All patients had appropriate wound pressure off-loaded, but we have no direct information on patient compliance with this in the outpatient setting.

### 3.2. Gram-Staining and Microbiological Cultures

We recorded 99 different bacterial culture constellations according to criteria of the Clinical Laboratory Standards Institute (CLSI (previously)) and the European Committee on Antimicrobial Susceptibility Testing (EUCAST (new)) [20], while the Gram-staining procedures remained unchanged throughout the study protocol. All Gram-staining was performed by professional microbiologists (and their laboratory assistants) in the University of Zürich’s IMM (Institut für Medizinische Mikrobiologie (IMM)). Results of wound cultures were available 1–3 days after sampling, at least for antibiotic-naïve DFIs. The microbiological laboratory did not report Gram stain results in 74% of cases (914/1235). Among the 321 (26%) for which Gram stain results were reported, the microbiologists did not visualize bacteria in 46% of episodes (149/321). Thus, useful Gram-staining results were available in only 54% of the cases. Among those with reported Gram-staining, we colored microorganisms in 118 episodes (118/321; 37%). There was no statistically significant difference between episodes with and without preoperative antibiotic administration (118/113 (51%) versus 54/36 (60%) with a 95% confidence interval 0.4–1.2, *p* = 0.15).

Overall, 35% of all DFI cultures were polymicrobial. Among isolated pathogens, 25% were Gram-negative bacteria. The eight most frequently-cultured pathogenic organisms or groups from all cases were as follows: coagulase-negative staphylococci (n = 258), *Staphylococcus aureus* (224), enterococci (60), *Pseudomonas* spp. (52); streptococci (33); *Escherichia coli* (31); *Proteus* spp. (23); and, obligate anaerobes (17). Among the cases in which organisms were visible on the Gram-stained smear, the main pathogen groups were quite different: *S. aureus* (n = 77); enterococci (33); coagulase-negative staphylococci (32); obligate anaerobes (24); *Pseudomonas* spp. (13); *Proteus* spp. (13); streptococci (12); and. *E. coli* (12). Specifically, coagulase-negative staphylococci were less frequently noted on the Gram stain, while *S. aureus* was the best detected. We do not know if they were less often seen, or less often reported (as technicians may have considered them non-pathogens). For cultures that grew *P. aeruginosa*, Gram-negative organisms were noted on the Gram-stained smears in only one-quarter of cases.

**Figure 1:**
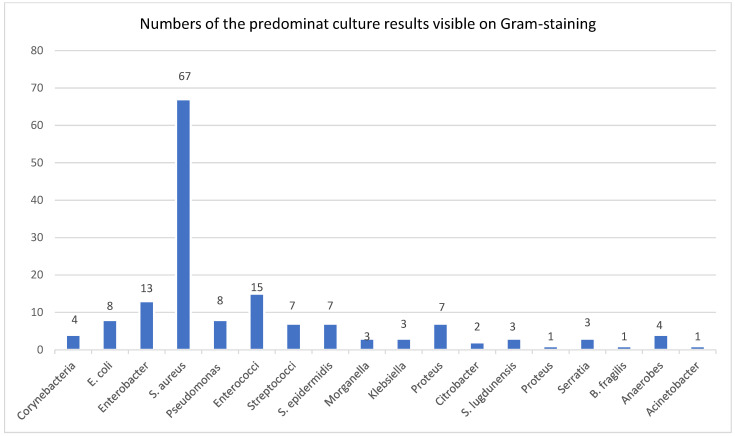
Predominant pathogen groups seen on the Gram-stained smear.

Among the Gram-stain smear on which organisms were detected, in 40% (69/172) of cases the organisms were typically resistant to our standard empirical antibiotic choice of amoxicillin/clavulanic acid, 40 of which were non-fermenting Gram-negative rods. Figure 1 displays the distribution of pathogen groups, which were visible on the Gram stain, in graphical form.

A pathogen was seen on an intraoperative Gram stain smear in 172 of 1235 cases (14%), 101 (59%) of which were only Gram-positive, 32 (19%) only Gram-negative, and 39 (33%) had both. Table 1 summarizes the concordance between the culture results and a prior Gram-stained smear (interpreted by an infectious diseases physician (I.U).

### 3.3. Prediction Performances of Cultured Pathogen Groups by the Gram Stain

Compared to the culture results, the statistical performance characteristics of the Gram-stained smear for Gram-positive organisms were as follows: sensitivity 56%; specificity 93%; accuracy 71%, positive predictive value 97%; and negative predictive value 38%. The corresponding values for Gram-negative organisms were as follows: 61%; 97%; 90%, 50%; and, 82%. When stratified for episodes under current (preoperative) antibiotic treatment, the combined values were 67%, 96%, 85%, 97%, and 49%. When stratified for monomicrobial infections only, these performances were 51%, 92%, 86%, 93%, and 48% for Gram-positive cultures, and 52%, 98%, 97%, 88%, and 90% Gram-negative cultures. Table 2 shows the links of the Gram stain results to the cultures yielding non-fermenting Gram-negative rods.

## 4. Discussion

In our setting of a large Swiss city hospital, the results of routine Gram stain smears of intraoperative DFI samples revealed a relatively low sensitivity throughout all of the investigated strata in predicting the pathogens found on cultured specimens. Overall, the Gram-stained smears reported pathogens only in half of the specimens from patients with moderate or severe DFI. Of note, many of the patients had been treated empirically with antibiotics before surgery. The pathogen most often visualized on the Gram-stained smears, as well found growing in the corresponding cultures, was *S. aureus*. The overall negative predictive values of the Gram-stained smears were moderate at best, and only reasonably clinically useful for monobacterial Gram-negative rods. We achieved the best accuracy with 97% for monomicrobial Gram-negative cultures. Of note, the positive and negative predictive values of the Gram-stained smears, as well as their statistical accuracies, largely depended on the local epidemiology of DFI pathogens, which might certainly be different in other centers [19]. When bacteria are observed in high numbers on the Gram-stain, the positive predictive value can be quite high. On the other hand, the negative predictive value is lower, which means that a negative Gram-stain result does not definitively exclude a bacterial infection. Negative Gram-stain results do not exclude the possibility of infection, and culture methods remain essential for identifying specific pathogens and determining their antibiotic susceptibilities. This discrepancy is especially important in cases where the bacterial load is low or the infection is in its early stages. Clinicians should interpret Gram-stain results in conjunction with clinical findings and consider specimen quality to make informed decisions about patient management.

The published literature on the clinical value of Gram-stained smears is sparse and dominated by three publications: the Abbas trial performed in Dar es Salaam, Tanzania [3]; the Taniguchi study from Okinawa, Japan [7]; and, the Chisman study from Birmingham, England [14]. Data from all three publications favor using the Gram stain smear in the initial management of DFI and other infections [4]. While the potential use of Gram-stained smear for assessing the likely causative pathogens in DFI is mentioned in some other articles (e.g., [21,22,23,24,25]). The Abbas paper is the most favorable for the use of the Gram stain, at least for a resource-limited setting in the less-developed world where availability of microbiological cultures is often limited. Both the available literature and our own experience suggest that using the results of a Gram-stained smear is better than just available clinical (and other laboratory) evidence for tailoring the empirical antibiotic choice. In particular, it is likely to reduce unnecessary prescribing of anti-Gram-negative coverage in regions already dealing with a high prevalence of multidrug resistance among these frequent pathogens [4,5,11,12].

The 2017 Chisman paper is the most similar to our study [14]. The authors investigated the role of microscopy in the management of DFI [14]. Among 114 tissue samples included, there were either no organisms or no predominant organisms seen in 50% of Gram stain smears. Gram stain results were in complete agreement with the final culture results only in 46%. Similar to our findings, 72% of the patients were already receiving systemic antibiotic agents at the time of sampling. Also, as in our study, the overall sensitivity and specificity of the Gram stains were relatively low (75% and 70%, respectively), but the specificity for Gram-negative pathogens was almost 99%. The authors concluded that while the Gram stain smear was a poor predictor of culture results, because it was quick and inexpensive it had a role in investigating the microbiology of DFI. Furthermore, they speculated that the presence of Gram-negative rods was specific enough to allow early broadening of the empirical antibiotic regimen [14], but did not provide evidence for this in their treatment outcomes. In that sense, we confirm all findings of the 2017 Chisman paper. The difference is that this paper might consider using the Gram stain for an anti-Gram-negative antibiotic broadening, whereas we investigate if it could reduce the recommended spectrum.

While there are certainly potential benefits to obtaining a Gram-stained smear to help in selection of empiric therapy, there remain some problems. These include the possibility of missing non-predominant or poorly visualized co-pathogens in a polymicrobial DFI, and the lack of being able to confidently identify the particular species of pathogen based solely on its Gram stain appearance [4]. For example, a Gram-negative rod could be a susceptible *Proteus* spp., or an *Enterobacter* spp. naturally resistant to many first-line β-lactam antibiotics, which would require different antibiotic treatments. For this reason, and to determine the antibiotic susceptibilities of visualized organisms, cultures of specimens are still required. Another problem with the Gram-stained smear is that it requires at least a certain amount of inoculum, with an adequate load of bacteria, to see the microorganisms and, very probably, an antibiotic-naïve sampling. This is often not the case in paucibacillary mild DFI with minimal drainage, or in patients already on antibiotic therapy. Additionally, inter-observer variabilities in reading Gram-stained smears can be substantial, especially when comparing experienced clinicians or microbiologists versus junior house officers or medical students, who are now less often training in this skill than in the past. With fewer hospital clinical microbiology laboratories now performing their own Gram-stained smears, there are fewer opportunities for clinicians or microbiologists to attain or maintain proficiency.

Regarding selecting antibiotic regimens, in mild and moderate DFI, clinicians do not usually need to attempt to cover all pathogens in soft-tissue DFI from the start, especially if the patient undergoes surgical treatment [4,16]. For example, many moderate and ischemic DFI harbor obligately anaerobic organisms that may be secondary pathogens, but we often leave these to open air after debridement without administering specific anti-anaerobe antibiotics [4]. In accordance with international guidance [1], we usually concentrate on covering the one or two most virulent pathogens, assuming that additional isolates found on culture are either non-virulent colonizers or contaminants. This approach, however, requires having adequate clinical and microbiological experience, and careful assessment of the clinical response to the empiric therapy. After initiating empiric antibiotic therapy, the choice of the definitive regimen must consider the patient’s clinical response, as well as the local epidemiology and antibiotic susceptibilities of pathogens [4]. The Gram stain smear also shares an important shortcoming of cultures. When more than one type of organism is seen or grown, especially from macerated or necrotic areas, neither can definitively distinguish between true pathogens and colonization/contamination. For example, in a similar setting of superficial burn infections, studies have demonstrated only a fair degree of microbiological correlation between the Gram stain and the pathogens of clinical infection (kappa statistic 0.32) [26].

While we think there is practical value in obtaining Gram-stained smears for selecting empiric antibiotics for treating a DFI, especially in resource poor settings, their usefulness (especially in resource-rich settings) are still mostly hypothetical. We lack well-designed prospective trials assessing the effectiveness of Gram-stained smears in improving antibiotic therapy and clinical outcomes. The conclusions of the Chisman study [14] suggested using the Gram-stained smear could allow early broadening of antibiotic treatment (if Gram-negative rods are seen), rather than the narrowing aimed at by antimicrobial stewardship. In resource-rich settings, one potential design might be investigating the potential usefulness of the Gram-stained smear in a study limiting the empiric antibiotic spectrum for mild and moderate DFI cases, while administering broad-spectrum agents for severe infections, as recommended in the IWGDF/IDSA guidelines [1,2]. For individual episodes, this benefit might be small, but from a large-scale societal viewpoint, even a short time period of avoidance of a broad-spectrum therapy could still be beneficiary. When performing prospective trials, the quality of the tissue sampling is key in all trial considerations. We cannot emphasize enough that the Gram-smear quality grading will always largely depend on the sampling approach [27]. We emphasize the necessity for a prospective trial design. An retrospective alternative will not provide the necessary in-depth documentation regarding past decisions about choosing empirical regimens. Moreover, it will be substantially biased by “confounding by indication” inherent to retrospective evaluations of medical interventions, especially within a setting with multiple concomitant randomized trials in DFI management [28]. Such a retrospective analysis would only be meaningful in a setting naïve to other interferences of “antibiotic stewardship” [18].

## 5. Conclusions

While Gram-stained smears offer immediate insights and can guide initial antimicrobial therapy in soft-tissue DFI, they should not be solely relied upon for definitive diagnosis. The Gram stain is probably very useful in the management of DFI and regarding antibiotic stewardship in many resource-poor settings with limited access to microbiology, a high prevalence of Gram-negative pathogens, and difficulties for surgery. Based on an evaluation of data from our urban Swiss setting, and the sparse literature in temperate developed regions [4,7,14], the results of the Gram stain smear from deep intraoperative DFI tissue samples provided limited usefulness for the prediction of cultured pathogens in major clinical strata investigated. The overall negative predictive values for the Gram-stained smears were unsatisfactory, and only reasonably acceptable for the subgroup of cases caused by Gram-negative rods. Prospective trials are needed to determine the value of the Gram-stained smear in supporting antibiotic stewardship in resource-rich settings, such as in Switzerland. Such trials would be pragmatically difficult, and their results likely to provide benefits that would be more epidemiological than clinically helpful to patients.

## Figures and Tables

**Table 1:** Crude associations of Gram-stained smear and final bacterial culture reports.

Visible Bacteria, n = 172	Gram-Positive Culture *	Gram-Negative Culture *	Proportion Mixed Cultures °
Gram-positive (n = 101)	High concordance (n = 99; 95%)	Incorrect concordance (0; 0%)	Minority (19; 19%)
Gram-negative (n = 32)	Low concordance (n = 3; 9%)	Correct concordance (29; 91%)	Minority (11; 34%)
Mixed (n = 39)	Moderate concordance (31; 79%)	Correct negative (34; 87%)	Correct both (31; 79%)

* in relation to bacterial cultures results; ° mixed cultures in terms of the Gram classification of the pathogens.

**Table 2:** Gram stain regarding monobacterial, non-fermenting Gram-negative infections.

Gram Stain Results	Culture of With Non-Fermenting Gram-Negative Bacteria	Culture with Gram-Positive Bacteria	Total
Gram-positives	10 (false-negative)	91 (true-negative)	101
Gram-negatives	30 (true-positive)	11 (false-positive)	41
Total	40	102	142

Sensitivity 0.75, Specificity 0.89, Positive predictive value 0.75, Negative predictive value 0.90.

## Data Availability

Key data are available in an anonymous form upon reasonable scientific request to the corresponding author. They are not publicly available.
